# Exploring Occupational Therapy Interventions for Heatstroke in Emergency and Critical Care Settings: A Retrospective Study

**DOI:** 10.7759/cureus.84836

**Published:** 2025-05-26

**Authors:** Masayoshi Seki, Takuya Maeda, Risa Mizushima, Fumihito Kasai

**Affiliations:** 1 Rehabilitation Center, Koto Toyosu Hospital, Showa University, Tokyo, JPN; 2 Physical Therapy, School of Nursing and Rehabilitation Sciences, Showa University, Tokyo, JPN; 3 Rehabilitation Center, Fujigaoka Rehabilitation Hospital, Showa University, Yokohama, JPN; 4 Rehabilitation Medicine, School of Medicine, Showa University, Tokyo, JPN

**Keywords:** activities of daily living (adl), emergency medicine, heatstroke, occupational therapy, rehabilitation, severe illness

## Abstract

Background

Heatstroke is a life-threatening condition, the incidence of which has been exacerbated by climate change and urbanization. Despite its increasing prevalence, rehabilitation for managing heatstroke, particularly occupational therapy (OT), remains underexplored.

Objective

This study aimed to investigate the implementation, characteristics, and effects of OT in patients hospitalized with heatstroke who were admitted to the emergency department.

Methods

We conducted a retrospective analysis of 49 patients diagnosed with heatstroke who were admitted to an emergency critical care center between July and September from 2019 to 2022. The patients were classified according to severity using the Japanese Association for Acute Medicine Heatstroke (JAAM-HS) criteria. Data on demographics, clinical outcomes, and OT interventions were collected. Statistical analyses included t-tests, chi-square tests, logistic regressions, and linear regressions.

Results

Among the 49 patients (median age: 72 years), 20 were classified as Grade II and 29 as Grade III. OT was primarily implemented in severe (Grade III) cases and focused on training in activities of daily living, neurological rehabilitation for cerebellar ataxia, and mobility support. Patients with Grade III heatstroke had significantly worse discharge outcomes (p = 0.006) and were more likely to be transferred to another facility. OT participation was not significantly associated with discharge destination but was associated with longer hospital stays (p = 0.003), likely reflecting greater illness severity. Age and sex were not significant predictors of the outcome.

Conclusion

This study highlights the variable implementation of OT in patients hospitalized with heatstroke and underscores the need for standardized rehabilitation protocols. Considering the potential for long-term neurological sequelae in severe cases, early and structured OT may support functional recovery. Further prospective studies are warranted to investigate the potential effectiveness of OT interventions and to develop evidence-based strategies tailored to illness severity.

## Introduction

Heatstroke is a severe heat-related illness characterized by hyperthermia (core body temperature >40.5°C), central nervous system dysfunction, and multi-organ failure. It is broadly classified into classic and exertional heat stroke, which affect vulnerable populations and physically active individuals, respectively [1-3]. The increasing frequency of heat waves due to climate change has exacerbated the risk, particularly in urban areas where heat stress has intensified, as demonstrated by the 2003 heat wave in Lyon, France [4].

The International Labor Organization (ILO) has highlighted the rising global impact of heat stress, particularly among workers exposed to extreme temperatures. The ILO 2024 report emphasized that excessive heat presents year-round occupational health challenges, making heatstroke a critical public health concern. Addressing heatstroke from both medical and societal perspectives is essential to safeguard vulnerable populations and ensure occupational safety [5].

In Japan, heatstroke is diagnosed based on the Japanese Association for Acute Medicine Heatstroke (JAAM-HS) criteria [6,7], whereas Western countries use the Bouchama Heatstroke (B-HS) criteria [1], which include core temperature as a diagnostic factor. The JAAM classification categorizes heatstroke severity into three grades: Grade I (mild) presents with symptoms such as dizziness or fainting that do not require hospitalization; Grade II (moderate) requires hospital transfer due to symptoms such as headache, nausea, and fatigue, whereas Grade III (severe) necessitates intensive care for neurological impairment, seizures, or motor dysfunction. In this study, patients were classified according to the JAAM criteria [8]. The pathophysiology of heatstroke involves systemic inflammatory responses, increased gastrointestinal permeability, and multi-organ dysfunction. Neurological complications, including cerebellar ataxia and cognitive impairment, may persist long after recovery [9-11].

Early rehabilitation is essential for mitigating long-term consequences, such as disuse syndrome and aspiration pneumonia; however, standardized rehabilitation protocols remain scarce, necessitating further research.

Given the rising incidence of heatstroke and its potential long-term morbidity, this study examined the implementation of occupational therapy (OT) in patients hospitalized with heatstroke. By analyzing the frequency, characteristics, and types of OT interventions, this study aimed to clarify the current state of OT in emergency and critical care settings.

Although early rehabilitation is well recognized in intensive care settings, the role of OT in emergency heatstroke management remains poorly understood. There is a lack of standardized OT protocols for patients with heatstroke, particularly in the emergency and critical care settings.

## Materials and methods

Study design

This retrospective study was conducted at an emergency critical care center. Medical records of patients with heatstroke admitted between July and September from 2019 to 2022 were analyzed. This study aimed to identify the frequency and characteristics of OT interventions in patients hospitalized with heatstroke.

Participants

A total of 49 patients diagnosed with heatstroke were included in this study. The inclusion criterion was hospitalization owing to a primary diagnosis of heatstroke. The exclusion criterion was patients suspected of having heatstroke but later diagnosed with hyperthermia due to other medical conditions.

Data collection

The following variables were extracted from patient records: demographics (age, sex, and medical history); clinical severity and diagnosis; duration of hospital and intensive care unit (ICU) stay; functional status at discharge; OT interventions (e.g., activities of daily living (ADL) training, neurological rehabilitation such as balance and coordination exercises, cognitive therapy for delirium, and mobility support for patients with impaired consciousness); and outcomes (e.g., discharge destination and mortality).

Statistical analysis

All statistical analyses were performed using the appropriate statistical software. Descriptive statistics were calculated for continuous variables (mean ± standard deviation or median (interquartile range)) and categorical variables (frequency and percentage). The following statistical tests were conducted:

Comparison of Severity Groups (Grade II vs. Grade III)

Continuous variables (e.g., age and length of hospital stay) were analyzed using independent t-tests for normally distributed data and the Mann-Whitney U test for non-normally distributed data. Categorical variables (e.g., sex, discharge outcome, and rehabilitation participation) were analyzed using the chi-square test or Fisher's exact test, as appropriate.

Types of OT Interventions

Discharge destination (home vs. other (transfer, death)): Logistic regression was performed with rehabilitation participation as the independent variable after adjusting for severity and age.

Length of hospital stay: The Mann-Whitney U test was conducted to compare the hospital stay duration between patients with and without rehabilitation.

Effect of Age (65 Years and Older vs. Younger Than 65 Years)

To assess the effect of age, patients were divided into two groups: those aged 65 years and older and those under 65 years. Chi-square tests were used to compare discharge destinations and rehabilitation participation between these groups. The Mann-Whitney U test was applied to compare hospital stay duration.

A p-value of less than 0.05 was considered statistically significant.

## Results

The total number of participants was 49, consisting of 14 females and 35 males. The median age of all participants was 72 years, with a wide age range from 13 to 94 years. Based on the JAAM-HS classification, 20 patients (40.8%) were classified as Grade II, while the remaining 29 patients (59.2%) were classified as Grade III.

Rehabilitation was initiated at the discretion of the attending physician. In general, patients with Grade III heatstroke were more likely to be referred for early rehabilitation, reflecting the need for intensive management. In contrast, referrals among Grade II patients varied according to individual clinical status, such as functional decline or prolonged fever.

Comparison of severity groups (Grade II vs. Grade III)

There were no statistically significant differences in age (p = 0.202) or sex distribution (p = 0.418) between the Grade II and Grade III groups. Although patients in the Grade III group tended to be older, the difference was not statistically significant.

As shown in Table 1, the median length of hospital stay was slightly shorter in the Grade III group (3.0 days) than in the Grade II group (4.0 days), but this difference was not statistically significant (p = 0.111). This may suggest that some Grade II patients had prolonged hospitalizations due to specific complications or social factors.

Regarding discharge outcomes, a significant difference was observed. Patients with Grade III heatstroke were significantly less likely to be discharged home (20.7%) compared to those with Grade II (65.0%; p = 0.006). Moreover, 72.4% of Grade III patients required transfer to another medical or rehabilitation facility, and 6.9% died during hospitalization. In contrast, no deaths occurred in the Grade II group. These clinical outcomes are summarized in Table 1.

**Table 1:** Comparison of clinical outcomes between severity groups Values are presented as median (interquartile range) or number (%). The chi-square test and Fisher’s exact test were used for categorical variables; the Mann-Whitney U test was used for continuous variables.

Comparison	Grade II (n = 20)	Grade III (n = 29)	Test statistic	p-value
Length of hospital stay (days)	4.0 (2.0-16.25)	3.0 (1.0-6.0)	U = 226.5	0.111
Discharge home (%)	13 (65.0%)	6 (20.7%)	χ² = 8.76	0.006
Transferred to facility (%)	7 (35.0%)	21 (72.4%)	χ² = 5.63	0.018
Mortality (%)	0 (0.0%)	2 (6.9%)	Fisher's exact test	0.507

Effects of age (65 years and older vs. younger than 65 years)

Table 2 presents patient characteristics, including age, sex, and heatstroke severity, stratified by age group (<65 vs. ≥65 years). There were statistically significant differences in age by definition (p < 0.001), but no significant differences in sex distribution (p = 0.844) or severity classification (p = 0.567) between the age groups.

**Table 2:** Patient characteristics and clinical profile Values are presented as median (range) or number (%). Mann-Whitney U test was used for continuous variables, and chi-square test was used for categorical variables. Statistical comparisons were conducted only on Grade II vs. Grade III as mutually exclusive categories.

Variable	Total (n = 49)	Age < 65 (n = 12)	Age ≥ 65 (n = 37)	Test statistic	p-value
Age (years)	72 (13-94)	45 (13-64)	78 (65-94)	U = 215.5	<0.001
Sex (male)	35 (71.4%)	11 (73.3%)	24 (70.6%)	χ² = 0.04	0.844
Severity - Grade II	20 (40.8%)	7 (46.7%)	13 (38.2%)	χ² = 0.33	0.567
Severity - Grade III	29 (59.2%)	8 (53.3%)	21 (61.8%)	-	-

As shown in Table 3, there was no significant difference in discharge destinations between the older and younger age groups (p = 0.208). Additionally, the length of hospital stay did not differ significantly between the two age groups (p = 0.127), suggesting that chronological age alone may not strongly determine clinical outcomes in this cohort.

**Table 3:** Comparison of clinical outcomes between age groups Values are presented as median (interquartile range) or number (%). Mann-Whitney U test was used for continuous variables, and chi-square test was used for categorical variables.

Comparison	Age < 65 (n = 12)	Age ≥ 65 (n = 37)	Test statistic	p-value
Length of hospital stay (days)	2.0 (1.0-4.25)	3.0 (2.0-10.0)	U = 143.5	0.127
Discharge home (%)	7 (58.3%)	12 (32.4%)	χ² = 1.59	0.208

Multivariable logistic regression analysis (predictors of discharge outcome)

The results of the multivariable logistic regression analysis are presented in Table 4. After adjusting for age, sex, and rehabilitation status, severity remained the only significant predictor of discharge to home.

**Table 4:** Predictors of discharge outcome - multivariable logistic regression analysis Multivariable logistic regression model adjusted for age, sex, occupational therapy, and severity. Odds ratio (OR) and 95% confidence interval (CI) are presented.

Variable	Odds ratio (OR)	95% CI	p-value
Severity (Grade III vs. Grade II)	0.159	(0.042-0.599)	0.008
Occupational therapy (yes vs. no)	0.786	(0.192-3.219)	0.729
Age (per year increase)	0.981	(0.944-1.019)	0.323
Sex (female vs. male)	0.375	(0.070-2.005)	0.252

Patients with Grade III heatstroke had significantly lower odds of being discharged home compared to those with Grade II (OR = 0.159, β = -1.84, p = 0.008), indicating a markedly worse prognosis. In contrast, participation in OT (OR = 0.786, p = 0.729), age (OR = 0.981, p = 0.323) and sex (OR = 0.375, p = 0.252) were not significantly associated with discharge outcomes in this model.

Multivariable linear regression analysis (determinants of hospital stay duration)

A separate multivariable linear regression analysis was conducted to examine factors associated with the length of hospital stay, and the results are shown in Table 5.

**Table 5:** Determinants of hospital stay duration - multivariable linear regression analysis Multivariable linear regression model adjusted for age, sex, occupational therapy, and severity. Regression coefficient (β) and 95% CI are presented.

Variable	Regression coefficient (β)	95% Cl	p-value
Severity (Grade III vs. Grade II)	-14.26	(-26.58, -1.94)	0.025
Occupational therapy (yes vs. no)	18.67	(6.23, 31.11)	0.003
Age (per year increase)	0.0406	(-0.234, 0.315)	0.798
Sex (female vs. male)	12.14	(-1.29, 25.57)	0.078

Patients with Grade III heatstroke had significantly shorter hospital stays compared to those with Grade II (β = -14.26 days, p = 0.025), likely reflecting more aggressive clinical decisions and early transfers in severe cases. Interestingly, those who received OT had significantly longer hospital stays (β = 18.67 days, p = 0.003), possibly due to rehabilitation being initiated in patients with persistent symptoms or delayed recovery.

No significant association was found between age and hospital stay (β = 0.041, p = 0.798). Although not statistically significant, female patients showed a trend toward longer hospitalization (β = 12.14 days, p = 0.078), which may warrant further investigation.

Occupational therapy in patients with heatstroke

*ADL* *Training*

ADL training consisted of targeted interventions aimed at home discharge (n = 8).

Neurological Rehabilitation

Neurological rehabilitation focused on cerebellar ataxia, with three patients requiring transfer to specialized recovery centers; rehabilitation strategies included balance training and fine motor skill exercises to address coordination deficits linked to Purkinje cell damage.

Cognitive Rehabilitation

Cognitive rehabilitation was provided to one patient through interventions focused on delirium management.

Mobility Training

Mobility training was applied to patients with severe consciousness disturbances or those requiring mechanical ventilation (n = 2).

## Discussion

This study investigated the characteristics and rehabilitation outcomes of patients hospitalized for heatstroke. These findings highlighted several important aspects of patient management.

Patients with Grade III heatstroke had significantly worse discharge outcomes than those with Grade II heatstroke, reinforcing the need for early intensive care. This finding is consistent with previous reports that exertional heatstroke frequently leads to multi-organ failure and poor clinical outcomes, particularly in severe cases [1,4]. However, despite having more severe disease, Grade III patients had shorter hospital stays than Grade II patients. This paradox could be explained by the higher rates of early transfer or mortality among Grade III patients (p = 0.006). In line with previous studies, severe cases of heatstroke often require transfer to specialized facilities owing to complex rehabilitation needs and long-term neurological impairments [12]. As our hospital functions as a tertiary emergency care center, many patients are transferred to rehabilitation or long-term care facilities once the acute phase treatment is completed. This institutional context may have contributed to the shorter hospital stays observed in some Grade III cases.

Prior studies have also shown that exertional heatstroke can lead to multi-organ failure, emphasizing the importance of early and intensive interventions to improve outcomes [1].

Furthermore, a study by Zeller et al. reported that patients with heatstroke who developed multi-organ failure had longer recovery times and worse functional outcomes, underscoring the need for specialized and continuous rehabilitation for these patients [12]. Therefore, this study supports previous research suggesting that exertional heatstroke can lead to multi-organ failure and prolonged hospitalization [12,13].

Effect of rehabilitation on patient recovery

Although patients who underwent rehabilitation had significantly longer hospital stays (p < 0.001), this was likely due to their greater severity at admission rather than the effect of rehabilitation itself. Previous research on critical care rehabilitation has shown that patients with more severe functional impairments are more likely to require intensive rehabilitation, contributing to prolonged hospital stays [1,4]. Additionally, rehabilitation did not significantly affect the discharge outcomes (p = 0.729). This may reflect the underlying severity of illness among patients who receive rehabilitation, as severe cases are more likely to require transfer to long-term care facilities. However, as this study was designed to describe the implementation of OT rather than to evaluate its effectiveness, causal inferences regarding discharge outcomes cannot be made. In contrast, less severe cases (Grade II) were more likely to recover sufficiently to be discharged without the need for extended rehabilitation interventions.

A systematic review reported that active mobilization and rehabilitation in ICU settings are associated with improved functional outcomes and reduced mortality [14]. Therefore, future studies should employ methods such as propensity score matching to control for severity differences and better assess the independent effects of rehabilitation on functional outcomes.

The role of occupational therapy in emergency heatstroke care

This study provides an overview of the use of OT in hospitalized patients with heatstroke. While OT was implemented in some cases, the criteria for referral and specific goals of the intervention varied. These findings highlight the need for clearer guidelines on OT implementation in emergency and critical care settings. Unlike stroke or traumatic brain injury, where structured rehabilitation protocols exist, OT interventions in heatstroke remain largely dependent on the treating physician’s discretion. In this study, OT interventions primarily focused on ADL training, neurological rehabilitation for ataxia, and mobility support for patients with consciousness disturbances. However, the variability in referral patterns indicates an urgent need for standardized rehabilitation guidelines for patients with heatstroke. Neurological impairments, such as cerebellar ataxia and cognitive dysfunction, were found to be common among Grade III patients who received rehabilitation. This aligns with prior reports indicating that heatstroke can cause long-term neurological damage, including the loss of Purkinje cells and cerebellar atrophy [9,10]. However, the criteria for OT referral remain unclear, highlighting the need for standardized rehabilitation protocols tailored for heatstroke-related complications.

OT brings a unique strength to critical care through its expertise in promoting functional independence and patient-centered interventions, particularly in training ADL and instrumental activities of daily living (IADL). Studies have shown that early OT involvement in ICU settings can significantly enhance functional recovery and reduce the negative impacts of prolonged ICU stays [11]. Moreover, recent scoping reviews highlight that OT interventions in ICUs not only support physical rehabilitation but also contribute to improved cognitive and psychosocial outcomes, underscoring the importance of integrating OT into multidisciplinary critical care teams [15].

Guidelines on rehabilitation of critically ill patients emphasize the importance of structured rehabilitation programs to improve functional outcomes and recovery [14]. However, no such guidelines currently exist for heatstroke rehabilitation. Developing evidence-based OT protocols tailored to heatstroke pathophysiology is essential for improving patient outcomes.

Age and functional outcomes

An age of 65 years or older did not significantly affect discharge outcomes or hospital stay duration. This finding contrasts with that of previous reports suggesting that older adults have a higher risk of heatstroke-related complications [2]. However, the relatively small sample size and the dominant effect of illness severity on age-related factors may have minimized the age-related disparities in this study.

Furthermore, early rehabilitation in ICU settings has been shown to be effective across different age groups, indicating that functional recovery may depend more on the severity of the heatstroke than on age itself [16]. This supports the notion that rehabilitation strategies should be tailored to the severity of illness rather than the patient’s age alone.

Comparison with existing literature

Previous studies have highlighted the importance of early rehabilitation in patients admitted to the ICU, although its specific impact on patients with heat stroke remains unclear. Our findings align with critical care rehabilitation research, which suggests that intervention strategies should be tailored to the severity of heatstroke rather than age alone [14].

Unlike stroke or traumatic brain injury, where structured rehabilitation protocols exist, OT interventions in heatstroke remain largely dependent on physician discretion. Previous studies have noted a lack of standardized rehabilitation protocols for patients with heatstroke, despite the well-documented benefits of early rehabilitation in critical care settings [1,4]. Developing evidence-based rehabilitation protocols tailored to the specific pathophysiology of heatstroke is essential to improve patient outcomes. The physiological response to heatstroke and the associated neurological impairments have been well described in previous studies [17,18]. The need for structured rehabilitation guidelines is supported by the demonstrated benefits of early mobilization and rehabilitation in ICU settings [14].

Limitations and future directions

The study was limited by its retrospective design and relatively small sample size (n = 49), which may have reduced statistical power, particularly for subgroup analyses such as age-based comparisons. This limitation may have contributed to the non-significant results of the age-related analyses.

In addition, detailed information on confounding factors such as baseline functional status, clinical frailty, and comorbidities was not consistently available in the medical records, limiting our ability to adjust for these factors in the analyses. Although physical therapy interventions were often provided and recorded alongside OT, this study focused exclusively on OT. Therefore, data on the frequency or content of physical therapy were not analyzed, which may have influenced the interpretation of rehabilitation effects.

Future research should include larger multicenter cohorts to improve the statistical robustness and validate these findings. Additionally, prospective studies evaluating individualized rehabilitation protocols for patients with severe heatstroke are necessary to optimize treatment strategies and to clarify the specific contribution of OT to functional recovery.

## Conclusions

This study highlighted the characteristics of OT implementation in hospitalized patients with heatstroke. These findings indicate that OT was administered primarily to severe cases, particularly to those with neurological impairments. However, the criteria for OT referral vary, emphasizing the need for standardized protocols for OT interventions in emergency and critical care settings. Given the global increase in heatstroke cases due to climate change, the development of standardized rehabilitation strategies is crucial.

Future research should focus on developing evidence-based rehabilitation protocols tailored to patient severity and evaluating long-term functional outcomes after discharge. This study highlights the need for a multidisciplinary approach to heat stroke management that integrates early intensive care and rehabilitation to improve patient outcomes.
